# Patient Perspectives on the Current Paradigm of Non-Invasive Cardiac Testing Through an Emergency Department Observation Unit: A Survey Study

**DOI:** 10.7759/cureus.85296

**Published:** 2025-06-03

**Authors:** Joshua C Reynolds, John A Oostema, Charles Sierzant, Kyle Sherwin

**Affiliations:** 1 Emergency Medicine, Michigan State University College of Human Medicine, Grand Rapids, USA; 2 Emergency Medicine, Corewell Health, Grand Rapids, USA; 3 Observation Medicine, Corewell Health, Grand Rapids, USA

**Keywords:** alternative care pathways, cardiology, chest pain, cross-section, emergency medicine, observation medicine, patient-centered outcomes, patient experience, survey

## Abstract

Background: Emergency department observation units (EDOU) facilitate cardiac testing after initial ED evaluation for acute coronary syndrome. While this approach offers multiple safety and compliance benefits, it also poses patient-facing costs and inconveniences that outpatient approaches might alleviate. Despite the clinical emphasis on shared decision-making when deciding to pursue further testing in the EDOU or ambulatory care setting, little is known about patient preferences on the matter. To inform the selection and design of care delivery models for patient-centered comparative analysis, we aimed to understand patient perspectives on their experiences undergoing cardiac observation and testing in our EDOU.

Methods: This iteratively developed and refined cross-sectional survey study enrolled a convenience sample of adults in an EDOU awaiting cardiac test results. Subjects completed a survey that included questions regarding expectations, experiences, quality of life, and perceived advantages/disadvantages of the EDOU stay. Subjects scored items as binary, numerical, and categorical responses; Likert scales; satisfaction ratings; and voluntary free text. We abstracted supplemental clinical data from the electronic health record. The primary outcome was binary willingness to participate in a hypothetical alternative outpatient testing program in lieu of the EDOU. After stratifying subjects by the primary outcome, we tabulated subject characteristics, survey responses, and subsequent clinical courses for pairwise comparison with t-tests, Wilcoxon rank-sum tests, chi-square tests, and Fisher’s exact tests.

Results: Of 100 subjects, 61% were willing to participate in a hypothetical outpatient program. Subjects willing to participate in the outpatient program had similar demographics, comorbidities, cardiac risk profiles, test results, cardiac procedures, and subsequent hospital courses to those who were not willing. Subjects valued the perceived benefits of rapid testing and safety in the EDOU but acknowledged the perceived drawbacks of financial cost and the need to make alternative arrangements in their absence from home. Subjects' perceived convenience of the hypothetical outpatient program was the strongest predictor of willingness to participate.

Conclusions: In this convenience sample of EDOU patients, subjects valued the timeliness and safety of hospital-based testing but noted the financial and opportunity costs. Most were willing to participate in a hypothetical outpatient testing program, but this was contingent on its perceived convenience. These findings inform the selection of patient-centered outcomes for comparative analysis of different care models to pursue additional cardiac testing after ED evaluation for acute coronary syndrome.

## Introduction

For emergency department (ED) patients with symptoms suggestive of acute coronary syndrome, ED observation units (EDOU) often facilitate additional cardiac testing for patients at intermediate risk of major adverse cardiac events [1-3]. Benefits of this approach include ensured compliance with testing, rapid turnaround, access to specialists, and close monitoring for adverse events. Yet EDOU stays do incur financial costs and inconveniences [4]. In light of rising healthcare costs, patients and payors are increasingly interested in leveraging shared decision-making [5] and telemedicine [6] to achieve superior patient-centered outcomes [7], cost-effectiveness, and value [8]. Novel care delivery models such as virtual observation medicine [9,10] or strategically deployed rapid access clinics [11,12] may provide alternative mechanisms for expedited outpatient testing for some patients who are currently placed in EDOUs [13]. Given acceptable safety parameters, these alternative mechanisms stand to offer favorable cost savings and patient satisfaction compared to hospital-based testing. Yet little is known about patient preferences regarding the optimal setting for workups of potentially serious diagnoses such as coronary artery disease. Such patient preferences are critical to selecting and designing care delivery models acceptable to patients and patient-centered outcomes for prospective assessment of comparative effectiveness between care delivery models.

To inform patient-centered features and outcomes pursuant to a new care delivery model, we aimed to understand patient perspectives on their experiences undergoing cardiac observation and testing in an EDOU. We surveyed a convenience sample of EDOU subjects to 1) describe patient-reported experiences, attitudes, and opinions about their EDOU stay, 2) identify which potential benefits and burdens of EDOU-facilitated testing are important to patients, and 3) determine factors associated with their willingness to participate in a hypothetical outpatient program. 

## Materials and methods

The Corewell Health Institutional Review Board approved this minimal-risk study. Subjects provided written informed consent at the time of study enrollment. This study was conducted according to the Checklist for Reporting of Survey Studies (CROSS) checklist [14].

Study design and population

This was a cross-sectional survey study of adult patients placed in an ED observation unit (EDOU) for additional cardiac testing between October 3, 2023, and June 19, 2024. Our EDOU is staffed by 15 board-certified emergency physicians with fellowship training and/or expertise in observation medicine, along with 13 experienced advanced practice providers. This EDOU is a complex, protocol-driven, Type 1 observation unit [15] that manages approximately 5,000 patients annually with a median length of stay of 24 hours. The EDOU cares for patients with a variety of diagnoses, of which chest pain is the most common. Overall, 5-10% of EDOU patients convert to inpatient status, and the remainder are discharged, typically within 23 hours. The EDOU serves a tertiary care center ED (approximately 100,000 adult visits annually) and provides weekend access to non-invasive cardiac testing for eight other community EDs in the same health system.

We approached a convenience sample of adult patients in the EDOU to enroll in this study. We excluded minors (age < 18 years), prisoners, subjects unable to provide written informed consent, and subjects who had already received the results of their cardiac testing. The survey instrument (Appendix) contained 46 questions distributed over seven sections that included a variety of question types (selection, Likert scale, rank-order, open-ended free-text) developed by the investigators iteratively through rounds of internal pilot testing and consensus discussion with refinement to map survey domains to the stated aims. Survey data were collected and managed using HIPAA-compliant REDCap subject-facing survey tools hosted at Corewell Health [16,17]. Paper copies of the consent form and survey were available as backups to accommodate patient preferences and were manually entered into REDCap. Respondents scored survey items during a single session as binary responses with embedded skip-logic, numerical responses from a drop-down menu, categorical selections from a drop-down menu, Likert scales to indicate degrees of agreement or disagreement, satisfaction rating scales, and free-text responses.

Data sources

In addition to the self-reported survey data provided by subjects, two clinical research nurses abstracted clinical data from the electronic health record (EHR) (Epic, Verona, WI) into REDCap at the time of subject enrollment with real-time cross-checks of accurate data entry.

Study definitions and outcomes

Subjects completed surveys before receiving the results of their cardiac stress test or computed tomography coronary angiogram (CTCA). The survey solicited data regarding the type of residence, number of dependents living at home, median household income, and type of health insurance. All other demographic and clinical features (age, sex, race, ethnicity, comorbidities) were abstracted from the EHR. The survey included questions regarding patient expectations, experiences, and quality of life during the EDOU stay, as well as perceived advantages and drawbacks of EDOU-based testing in lieu of outpatient testing. They also asked about subjects’ willingness to participate in a hypothetical home-based observation program.

The study team abstracted cardiac test results and the subsequent hospital course, including any additional testing or procedures, from the EHR. Two study authors (CS, KS), who staff the EDOU, adjudicated non-invasive cardiac test results as low, moderate, or high risk using predefined criteria based on institutional practice. For stress tests, those interpreted as ‘low risk’ or ‘low risk based on Duke’s treadmill criteria’ were classified as low risk, those interpreted as evidence of infarction without reversible ischemia were classified as moderate risk, and those with reversible ischemia were classified as high risk. For CTCAs, evidence of <49% stenosis was classified as low risk, 50-69% stenosis was classified as moderate risk, and >70% stenosis or any stenosis with abnormal fractional flow reserve (FFR) analysis was classified as high risk. Adjudicating non-invasive cardiac tests yielded a complete inter-rater agreement.

The primary outcome was whether subjects were willing to participate in a hypothetical alternative clinical pathway in which they could return the next day for additional testing in lieu of staying in the EDOU.

Statistical analyses

As a first step in this line of inquiry, we targeted an a priori sample size of 100 subjects without a specific power calculation. Subjects meeting enrollment criteria were only eligible to participate in this study once. Study staff manually ensured that candidate subjects had not previously participated. We performed statistical analyses with STATA 15.1 (StataCorp, College Station, TX). We stratified subjects by the primary outcome and tabulated subject characteristics, survey responses, and the subsequent clinical course. We compared these variables with a t-test (Wilcoxon rank-sum test for nonparametric variables) and chi-square or Fisher’s exact test. Finally, two study authors (JCR, JAO) independently categorized and tabulated the collection of voluntary free-text responses pursuant to hypothesis-driven thematic elements with near-complete agreement; a handful of disagreements were resolved by consensus discussion. Any missing respondent data are reported as such without imputation.

## Results

**Figure 1:**
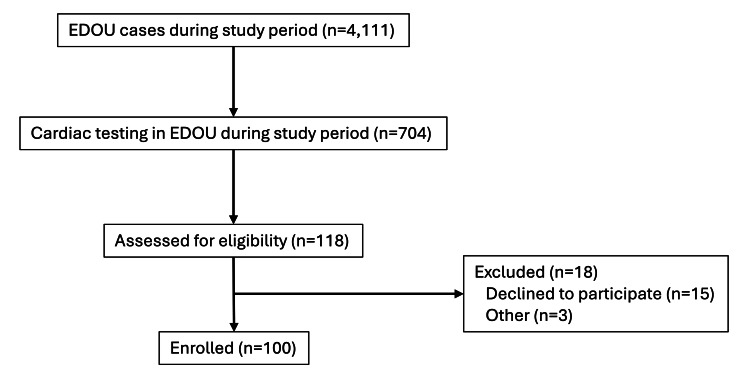
Study cohort and exclusions. EDOU: emergency department observation unit. The image is created by the author.

Among 4,111 adult patients placed in the EDOU during the study period, 704 had non-invasive cardiac testing (Figure 1). We screened 118 subjects and enrolled 100 (85% response rate). Of enrolled subjects, 61% indicated a willingness to participate in a hypothetical outpatient chest pain evaluation program.

Table 1 describes the demographic and clinical features of the cohort. Subjects had typical features for EDOU chest pain patients and reflected the underlying demographics of our region. This sample also reflects the population of interest: subjects with moderate HEART scores and equipoise about the optimal setting and care model in which to pursue additional cardiac testing after initial ED evaluation. Subjects who were willing to participate in an outpatient chest pain evaluation had similar demographics, comorbidities, and HEART scores to those who were not willing. Those willing to participate in an outpatient evaluation were less likely to have undergone prior cardiac testing (echocardiogram or stress test). Subjects who were willing to participate in outpatient testing were also more likely to be accompanied by a spouse/significant other.

**Table 1:** Demographics and clinical features of enrolled subjects. Data presented as count (percentage), mean ± standard deviation, or median (interquartile range). BMI: body mass index. PCI: percutaneous coronary intervention. CABG: coronary artery bypass graft. EHR: electronic health record. CTCA: computed tomography coronary angiogram. ED: emergency department. * p-values <0.05 are considered significant.

	All (n=100)	Willing to Return		X^2^ value	p-value
		No (n=39)	Yes (n=61)		
Demographics					
Age (years)	65.8 ± 10.7	67.4 ± 9.0	64.7 ± 11.6	-	0.22
Age categories (years)	-	-	-	1.4216	0.49
< 60	28 (28%)	10 (26%)	18 (30%)		
61 – 69	38 (38%)	13 (33%)	25 (40%)		
> 70	34 (34%)	16 (41%)	18 (30%)		
Female sex (%)	50 (50%)	21 (54%)	29 (48%)	0.3783	0.54
Race/Ethnicity	-	-	-	4.8125	0.31
Non-Hispanic White	87 (87%)	36 (92%)	51 (84%)		
Non-Hispanic Black	8 (8%)	1 (3%)	7 (12%)		
Hispanic ethnicity	3 (3%)	1 (3%)	2 (3%)		
American Indian/Alaska Native	1 (1%)	0 (0%)	1 (2%)		
No response	1 (1%)	1 (3%)	0 (0%)		
Type of Health Insurance	-	-	-	3.7552	0.44
Private	36 (36%)	12 (31%)	24 (43%)		
Medicare	28 (28%)	14 (36%)	14 (25%)		
Combination	23 (23%)	10 (26%)	13 (23%)		
Medicaid	7 (7%)	2 (5%)	5 (9%)		
Self-pay	1 (1%)	1 (3%)	0 (0%)		
No response	5 (5%)	0 (0%)	5 (8%)		
Median household income	-	-	-	5.7373	0.22
< $25,000	11 (11%)	4 (11%)	7 (13%)		
$25,000 to $50,000	31 (31%)	17 (47%)	14 (26%)		
$50,000 to $75,000	24 (24%)	9 (25%)	15 (28%)		
$75,000 to $100,000	15 (15%)	5 (14%)	10 (19%)		
> $100,000	8 (8%)	1 (3%)	7 (13%)		
No response	11 (11%)	3 (8%)	8 (13%)		
Living Situation	-	-	-	6.3286	0.27
Single family home	72 (72%)	28 (72%)	44 (72%)		
Condominium	9 (9%)	3 (8%)	6 (10%)		
Apartment	7 (7%)	3 (8%)	4 (7%)		
Mobile home	5 (5%)	4 (10%)	1 (2%)		
Multi-family home	4 (4%)	0 (0%)	4 (7%)		
Other / No response	3 (3%)	1 (3%)	2 (3%)		
Distance from residential zip code to hospital (miles)	11.7 (6.4 – 21.2)	15.6 (6.4 – 23.4)	11.1 (6.4 – 19.0)	-	0.37
Clinical Features					
BMI	32.1 ± 6.8	31.6 ± 7.2	32.4 ± 6.5	-	0.58
Comorbidities	-	-	-	-	-
Hypertension	60 (60%)	20 (51%)	40 (67%)	2.3432	0.13
Hyperlipidemia	61 (61%)	21 (54%)	40 (66%)	1.3754	0.24
Diabetes mellitus	26 (26%)	10 (26%)	16 (26%)	0.0043	0.95
Current tobacco use	15 (15%)	7 (18%)	8 (13%)	0.5128	0.47
Known coronary artery disease	31 (31%)	16 (41%)	15 (25%)	3.0043	0.08
Prior PCI	29 (29%)	13 (33%)	16 (26%)	0.5831	0.45
Prior CABG	6 (6%)	5 (13%)	1 (2%)	5.2734	0.03 *
Prior revascularization	31 (31%)	15 (38%)	16 (26%)	1.6641	0.20
Prior cardiac testing in the EHR	68 (68%)	31 (82%)	37 (61%)	4.7658	0.03 *
Echocardiogram (%)	59 (59%)	28 (74%)	31 (51%)	5.0835	0.02 *
Most recent EF (%) (n=59)	62 ± 8	61 ± 8	63 ± 8	-	0.27
Stress test (%)	57 (57%)	27 (71%)	30 (49%)	4.5858	0.03
Elapsed interval (years) (n=57)	3.7 (1.9 – 7.0)	3.7 (1.9 – 8.4)	3.6 (1.9 – 5.9)	-	0.51
CTCA	9 (9%)	4 (10%)	5 (8%)	0.1232	0.73
Elapsed interval (years) (n=9)	6.2 (1.5 – 11.8)	9.0 (3.8 – 12.6)	2.2 (1.5 – 9.0)	-	0.62
HEART score	-	-	-	1.9740	0.44
Low	7 (7%)	1 (3%)	6 (10%)		
Intermediate	85 (85%)	35 (90%)	50 (82%)		
High	8 (8%)	3 (8%)	5 (8%)		
Spent part of weekend in EDOU	31 (31%)	12 (31%)	19 (31%)	0.0016	0.97

Table 2 describes the hospital course for enrolled subjects. No subjects were transferred from an outside hospital ED for their EDOU stay. Most (67%) subjects received nuclear stress testing, 20% received an additional echocardiogram, 16% proceeded to invasive coronary angiography, and 10% had coronary revascularization. Subjects with a high-risk stress test or CTCA were more likely to proceed to invasive coronary angiography (85%) than those with low-risk (6%) or moderate-risk (7%) test results (p<0.001). Median EDOU length of stay was 19.3 (16.0 - 21.9) hours until either discharge or inpatient conversion. Subjects who were and were not willing to participate in an alternative clinical pathway had similar subsequent clinical courses, including EDOU length of stay, the likelihood of concerning test results, conversion to inpatient hospitalization, and cardiac procedures.

**Table 2:** Clinical course in the Emergency Department Observation Unit (EDOU). Data presented as count (percentage), mean ± standard deviation, or median (interquartile range). EKG: electrocardiogram. CTCA: computed tomography coronary angiogram. MRI: magnetic resonance imaging. PCI: percutaneous coronary intervention. CABG: coronary artery bypass graft. ICD: implantable cardiac defibrillator. * p-values <0.05 are considered significant.

EDOU clinical course	Whole Cohort (n=100)	Willingness to Return		X^2^ value	p-value
		No (n=39)	Yes (n=61)		
Non-invasive test performed	-	-	-	1.1652	0.87
Nuclear stress test	67 (67%)	28 (72%)	39 (64%)		
EKG stress test	1 (1%)	0 (0%)	1 (2%)		
Stress echo	18 (18%)	6 (16%)	12 (20%)		
CTCA	14 (14%)	5 (13%)	9 (15%)		
Test results	-	-	-	-	-
Stress test (n=86)	-	-	-	1.3544	0.51
Low risk	64 (67%)	23 (68%)	41 (79%)		
Moderate risk	12 (12%)	6 (18%)	6 (12%)		
High risk	10 (10%)	5 (15%)	5 (10%)		
CTCA (n=14)	-	-	-	2.9296	0.29
Low risk	8 (8%)	3 (60%)	5 (56%)		
Moderate risk	3 (3%)	2 (40%)	1 (11%)		
High risk	3 (3%)	0 (0%)	3 (33%)		
Combined (n=100)	-	-	-	1.5495	0.46
Low risk	72 (72%)	26 (67%)	46 (75%)		
Moderate risk	15 (15%)	8 (21%)	7 (11%)		
High risk	13 (13%)	5 (13%)	8 (13%)		
Conversion to inpatient admission	14 (14%)	5 (13%)	9 (15%)	0.0739	0.79
Cardiology consultation	24 (24%)	7 (18%)	17 (28%)	1.2835	0.26
Additional cardiac testing	-	-	-	-	-
Cardiac MRI	1 (1%)	0 (0%)	1 (2%)	0.6457	0.42
Echocardiogram	20 (20%)	6 (15%)	14 (23%)	0.8512	0.36
CTCA (following a stress test)	0 (0%)	0 (0%)	0 (0%)	n/a	n/a
Stress test (following a CTCA)	0 (0%)	0 (0%)	0 (0%)	n/a	n/a
Ambulatory cardiac monitoring (Zio^TM^ patch)	1 (1%)	1 (3%)	0 (0%)	n/a	0.21
Cardiac Procedures	-	-	-	-	-
Invasive coronary angiography (%)	16 (16%)	5 (13%)	11 (18%)	0.4808	0.49
PCI	6 (6%)	1 (3%)	5 (8%)	1.3383	0.25
CABG	4 (4%)	2 (5%)	2 (3%)	0.2119	0.65
Pacemaker/ICD	0 (0%)	0 (0%)	0 (0%)	n/a	n/a
Electrical cardioversion	0 (0%)	0 (0%)	0 (0%)	n/a	n/a
Chemical cardioversion	0 (0%)	0 (0%)	0 (0%)	n/a	n/a
EDOU length of stay (hours)	19.3 (16.0 – 21.9)	19.1 (15.9 – 21.4)	19.9 (16.4 – 22.5)	-	0.29
EDOU start-to-noninvasive test (hours)	14.9 (12.6 – 17.1)	14.8 (12.1 – 16.8)	15.0 (12.7 – 17.6)	-	0.53

Table 3 summarizes subjects’ attitudes, expectations, and quality of life for their EDOU stay. Most were accompanied by a friend or family member during their index ED visit and/or EDOU stay. Few expected to stay in the hospital when they presented to the ED, but subjects were typically worried about their symptoms and considered it important to pursue testing through the EDOU. Most subjects perceived a moderate sense of control over the decision to stay in the EDOU. Subjects visited by a spouse/significant other were more willing to participate in an alternative clinical pathway than subjects visited by an adult child. Subjects reported a generally favorable experience in the EDOU, with the notable exception of shorter duration of sleep with multiple interruptions and poor-to-moderate quality.

**Table 3:** Patient-centered context, expectations, and quality of life during the Emergency Department Observation Unit (EDOU) stay Data are presented as count (percentage) or median (interquartile range). ED: emergency department. * p-values <0.05 are considered significant.

	All (n=100)	Missing	Willingness to Return		X^2^ value	p-value
			No (n=39)	Yes (n=61)		
Context & Expectations						
Transferred from outside hospital ED	0 (0%)	3 (3%)	0 (0%)	0 (0%)	n/a	n/a
Somebody accompanied to ED visit	68 (68%)	0 (0%)	27 (69%)	41 (67%)	0.0445	0.83
Somebody accompanied to EDOU	57 (57%)	0 (0%)	22 (56%)	35 (57%)	0.0091	0.92
Visitor while in EDOU	45 (45%)	0 (0%)	19 (49%)	26 (23%)	0.3571	0.55
Spouse/significant other	20		2 (5%)	18 (30%)	8.8377	0.003 *
Friend	8		5 (13%)	3 (5%)	2.0186	0.26
Adult child	27		15 (38%)	12 (20%)	4.2612	0.04 *
Minor child	0		n/a	n/a	n/a	n/a
Other	6		4 (10%)	2 (3%)	2.0537	0.21
While in ED, expected to stay in the hospital?	23 (23%)	4 (4%)	11 (31%)	12 (20%)	1.3761	0.24
How worried about symptoms? (scale 0-10; 10 represents very worried)	7 (5-8)	2 (2%)	6 (5 – 8)	7 (5 – 9)	-	0.37
How important to stay in EDOU? (scale 0-10; 10 represents very important)	7 (5-9)	4 (4%)	5 (7 – 9)	7 (5 – 8)	-	0.05
How much control patient had over decision to stay in EDOU for more tests? (scale 0-10; 10 represents much control)	6.5 (4-9)	2 (2%)	7 (5 – 9)	6 (2.5 – 9.8)	-	0.57
Quality of Life during EDOU stay						
How many hours of sleep last night?	5 (3 – 6.5)	1 (1%)	5 (3 – 7)	5 (3 – 6.5)	-	0.62
How many interruptions in sleep?	2 (1-3)	6 (6%)	2 (1 – 3)	2 (1 – 3)	-	0.44
Sleep quality the prior night? (scale 0-10; 10 represents excellent quality)	5 (2 – 7)	2 (2%)	5 (3 – 7)	4 (1 – 7)	-	0.32
Did patient eat hospital food?	66%	1 (1%)	26 (67%)	40 (67%)	0.0000	1.0
Food quality? (n=66) (scale 0-10; 10 represents excellent quality)	5 (5 – 7)	1 (1.5%)	6 (5 – 7)	5 (4 – 6)	-	0.05
Cell signal quality (Likert 1-5)	-	5 (5%)	-	-	4.016	0.13
Excellent/Good	71 (71%)		26 (67%)	45 (74%)		
Neutral	15 (15%)		9 (25%)	6 (10%)		
Poor/Very Poor	9 (9%)		2 (5%)	7 (11%)		
Wi-Fi signal quality (Likert 1-5)	-	15 (15%)	-	-	0.5337	0.88
Excellent/Good	55 (55%)		19 (49%)	36 (59%)		
Neutral	21 (21%)		7 (25%)	14 (25%)		
Poor/Very Poor	9 (9%)		2 (7%)	7 (11%)		
Entertainment options (Likert 1-5)	-	7 (7%)	-	-	0.3101	0.90
Excellent/Good	58 (58%)		22 (56%)	36 (59%)		
Neutral	25 (25%)		10 (29%)	15 (26%)		
Poor/Very Poor	10 (10%)		3 (9%)	7 (11%)		
Freedom of movement (Likert 1-5)	-	3 (3%)	-	-	3.2915	0.19
Excellent/Good	69 (69%)		24 (62%)	45 (74%)		
Neutral	13 (13%)		8 (21%)	5 (9%)		
Poor/Very Poor	15 (15%)		6 (16%)	9 (15%)		

**Figure 2:**
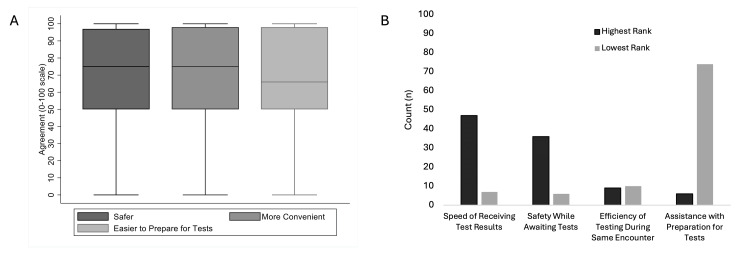
Subject-perceived benefits of EDOU stay for additional testing. A: Perceived benefits of EDOU-based testing in lieu of outpatient testing (higher rating corresponds to greater agreement). B: Rank order of candidate benefits of EDOU-based testing with proportions of highest and lowest assigned rank. The image is created by the author.

**Figure 3:**
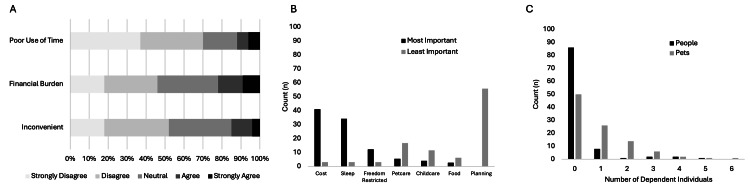
Subject-perceived drawbacks of EDOU stay for additional testing. A: Perceived drawbacks of EDOU-based testing in lieu of outpatient testing (Likert scale). B: Rank order of candidate drawbacks of EDOU-based testing with proportions of highest and lowest assigned rank. C: Number of individuals (persons and pets) for whom patients report being the primary caregiver. The image is created by the author.

When asked about the value of the EDOU, most subjects identified safety and convenience as the greatest advantages compared to outpatient testing. Subjects were less concerned about the efficiency of completing testing during the same healthcare encounter or the incremental value of immediately available assistance with preparatory instructions for testing (Figure 2). Few subjects considered the EDOU stay inconvenient or a serious financial burden, yet when asked about drawbacks to the EDOU, poor sleep, and cost were most identified. Of 12 general free-text responses, two subjects articulated the tradeoff between perceived safety while in the hospital versus the cost and convenience in considering the hypothetical outpatient evaluation alternative. A substantial number of subjects indicated that their pets required care in their absence (Figure 3). Of the 18 voluntary text responses specifying arrangements that subjects made to accommodate the EDOU stay, eight mentioned animal care, three mentioned dependent care, and one mentioned plant care.

**Figure 4:**
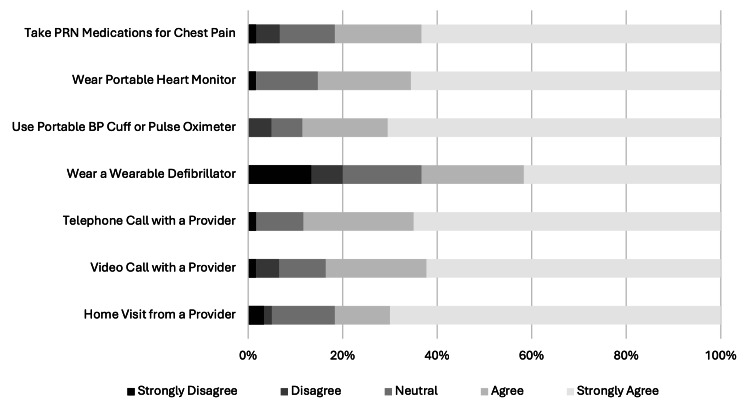
Subject willingness (Likert Scale 0-5) to utilize candidate components of a home-based observation medicine program for chest pain. PRN: as needed. BP: blood pressure. The image is created by the author.

Nearly half (43%) of subjects indicated that an outpatient evaluation program would be convenient or very convenient, while 30% were neutral, and 27% felt it would be inconvenient or very inconvenient. Those perceiving convenience in an outpatient evaluation program were much more likely to indicate willingness to consider it as an option (59% vs. 10%; p<0.001) than subjects perceiving inconvenience in the same outpatient program. Of the 27 subjects noting inconvenience to an alternative to EDOU-facilitated testing, the dominant reason (67%) was transportation concerns; however, driving distance from the residential zip code to the hospital did not differ between willing or unwilling subjects (Table 1). When asked about various forms of monitoring that would be acceptable at home (Figure 4), 61 subjects indicating willingness to participate in an alternative care pathway reported the least palatable component was a wearable defibrillator (38% disagreed or strongly disagreed). A small minority of subjects were opposed to the prospect of taking medications for returning symptoms (7% disagreed or strongly disagreed), wearing portable monitoring devices (2-5% disagreed or strongly disagreed), or various modalities of clinician check-in (2-7% disagreed or strongly disagreed).

## Discussion

These data provide patient-level insights into the experiences and opinions of patients admitted to an EDOU for a cardiac evaluation. While subjects in our survey recognized the need for rapid evaluations, the majority would be open to non-hospital-based approaches to accomplish chest pain workups.

Subjects were generally concerned about their symptoms and thought the EDOU stay was valuable, citing the perceived benefits of rapid testing and the perceived safety of being in a monitored setting in case of an adverse event. Most subjects had a favorable impression of their EDOU experience. One notable exception to this was the consistent report of poor sleep quality and frequent interruptions, which is a known problem for hospitalized patients [18,19]. Subjects also acknowledged the perceived drawbacks of an EDOU stay, noting both direct financial costs and indirect opportunity costs of making alternative arrangements in their absence from home. Nevertheless, we were surprised to find that cost concerns about the EDOU stay were not highly prevalent. We suspect that enrolled subjects may not have yet known the cost burden of their stay, despite the current healthcare trends toward price transparency [20-23].

More than half of the subjects surveyed were willing to participate in a hypothetical outpatient program for expedited cardiac testing. The perceived convenience of such an approach was the strongest predictor of interest in outpatient testing, whereas demographic features, socioeconomic features, medical risk stratification, and patient satisfaction with the EDOU stay were largely not associated with interest. The primary driving factor of perceived convenience was access to reliable transportation. On the other hand, prior cardiac testing was associated with lower interest in outpatient programs, suggesting that patients may be directed by their own perception of the risk of serious disease. Such perceptions that misalign with actual patient risk might be modifiable with tailored education.

Subjects were very receptive to the prospect of ambulatory monitoring or check-ins from a clinician. Most subjects (62%) were even willing to wear a portable defibrillator vest, but this was the least palatable component of home-based monitoring. Nonetheless, these findings illustrate a surprising degree of openness to leveraging technology at home to ensure safety [24]. The necessary intensity of home-based monitoring for an alternative care pathway is dictated by robust estimates of patient-level short-term risk of adverse events. Initial short-term risk estimates from a larger cohort of subjects in our EDOU suggest that these risks are very low [25].

Limitations and future opportunities

This sample is representative of the demographic and clinical features in our region and health system, which may limit generalizability to other locales. Our convenience sample is enriched with subjects with material coronary disease (10% revascularization rate) and thus may not apply to lower-risk populations. Given the nature of our study design, these data do not inform how discussions with clinicians regarding their risk of adverse events would influence patient preferences. Subjects enrolled in this study prior to receiving the results of their cardiac testing and likely prior to receiving hospital bills. It is possible that their self-assessment of risk and costs might be further influenced by this information, but this was an intentional aspect of study design that reflects circumstances in which patients would be approached to discuss outpatient alternatives for further evaluation. There are several opportunities to further advance this work. Cognitively interviewing patients and conducting psychometric analysis could validate this survey as a formal instrument conducive to multivariable modeling or causal inference. This instrument could then be administered before and after subjects receive their test results and/or hospital bills. 

## Conclusions

Among a convenience sample of adults in the EDOU awaiting results of cardiac testing, subjects valued the timeliness and safety of hospital-based testing but also noted the associated financial costs, opportunity costs, and necessity of making arrangements in their absence from home. Most subjects were willing to participate in a hypothetical outpatient cardiac evaluation program that provides expedited cardiac testing without an associated EDOU stay, but this willingness was contingent upon its perceived convenience, which was, in turn, primarily associated with transportation access. This willingness was discussed hypothetically with subjects, and actual patient behavior might vary due to external factors. These findings inform the design of care delivery models that are acceptable to patients and patient-centered outcomes for prospective assessment of comparative effectiveness between care models to pursue additional cardiac testing after initial ED evaluation for acute coronary syndrome. 

## Figures and Tables

**Figure 5:**
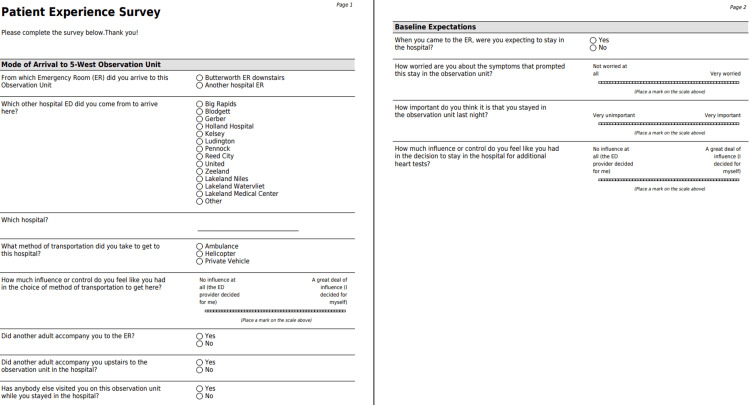
Survey pages 1-2

**Figure 6:**
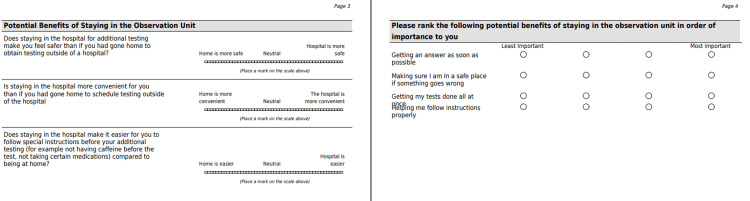
Survey pages 3-4

**Figure 7:**
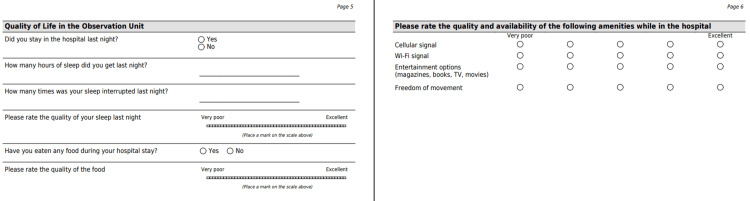
Survey pages 5-6

**Figure 8:**
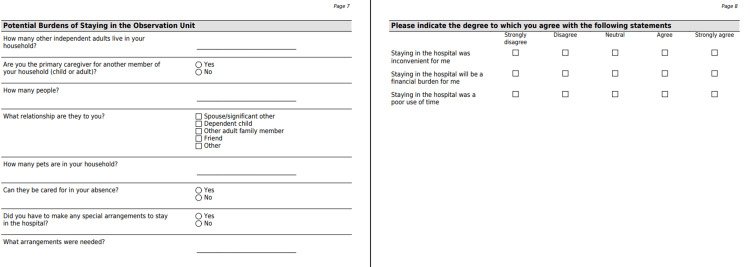
Survey pages 7-8

**Figure 9:**
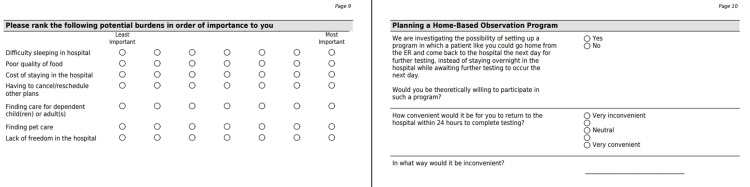
Survey pages 9-10

**Figure 10:**
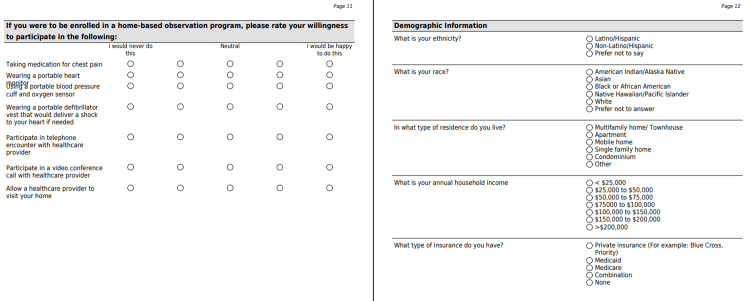
Survey pages 11-12

**Figure 11:**
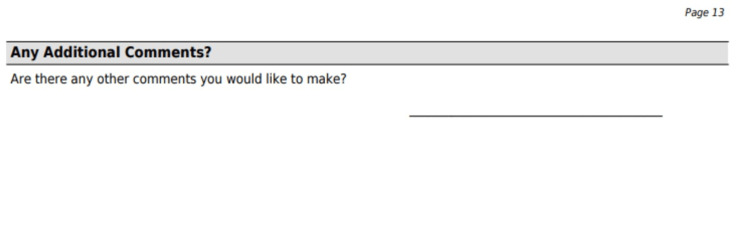
Survey page 13

## Appendices

Subject-facing survey
